# Influence of ageing on *longissimus lumborum* quality from Holstein-Friesian young bulls fed different diets

**DOI:** 10.1007/s13197-019-03778-7

**Published:** 2019-06-10

**Authors:** Monika Modzelewska-Kapituła, Katarzyna Tkacz, Zenon Nogalski, Mirosława Karpińska-Tymoszczyk, Adam Więk

**Affiliations:** 1 Department of Meat Technology and Chemistry, Faculty of Food Sciences, University of Warmia and Mazury in Olsztyn, Plac Cieszyński 1, 10-719 Olsztyn, Poland; 2 Department of Cattle Breeding and Milk Evaluation, Faculty of Animal Bioengineering, University of Warmia and Mazury in Olsztyn, Oczapowskiego 5, 10-719 Olsztyn, Poland; 3 Department of Human Nutrition, Faculty of Food Sciences, University of Warmia and Mazury in Olsztyn, Słoneczna 45F, 10-719 Olsztyn, Poland

**Keywords:** Beef, Cattle feeding, Meat, Tenderness, Wet ageing

## Abstract

The aim of the study was to investigate the effect of ageing (9 and 14 days) on beef obtained from Polish Holstein-Friesian bulls (n = 24) fed different dietary treatments containing the addition of herbal preparations (treatments: control, one and two herbal preparations). Between the 9th and 14th day of ageing, moisture and expressible water contents, Warner–Bratzler Shear Force (WBSF) and intensity of untypical taste significantly decreased, whereas redness, yellowness, chroma, intensity of meat aroma and tenderness increased. Interactions between ageing and dietary treatment on pH, expressible water, cooking loss and WBSF were noted. Using two herbal preparations in a dietary treatment enabled to obtain 9-days aged beef with similar tenderness as compared with 14-days aged beef from the control treatment. Therefore, it is possible to decrease the ageing time of beef by using a well-designed dietary treatment.

## Introduction

There are many factors which affect beef quality, e.g. cattle breed, sex, age, pre- and post-slaughter handling. In Poland, the most common cattle breed is Polish Holstein-Friesian (Iwanowska and Pospiech 2010), which is a dairy breed. Since the carcasses of the cattle show lower slaughter parameters (lower dressing percentage and degree of conformation) and meat quality (Bureš and Bartoň 2018) than beef or crossbred cattle (Węglarz 2010), different strategies for improving the quality have been introduced, e.g. using different dietary treatments (Nian et al. 2017a), fattening to higher slaughter weight (Węglarz 2010) or electrical stimulation of the carcasses (Li et al. 2011). On the other hand, there are also reports showing similar or even better eating quality of beef obtained from dairy breeds (Devlin et al. 2017), which indicates that meat obtained from dairy breeds might be valuable material for culinary applications.

A key factor which determines beef tenderness and other eating attributes is ageing (Nian et al. 2017b). It is well-established that during ageing, the tenderness of beef increases, which is caused by the changes in the myofibrillar structure due to the activity of endogenous proteolytic enzymes (Muchenje et al. 2009). Beef is usually aged for 7 to 21 days (Resconi et al. 2018). The minimal ageing period is included in the guidelines for producing high quality beef in certified systems. One of them is Quality Meat Program **(**QMP) which, since 2008, has been acknowledged by Polish Ministry of Agriculture and Rural Development as an official domestic beef quality system. The system requires at least 9 days of post-mortem ageing (PZPBM 2016). However, in the system, only beef from a particular beef breeds and crosses may be certified. On the other hand, the ageing period of 14 days is recommended by AMSA (2015) to study the tenderness of beef due to the fact that meat aged 14 days has acceptable tenderness and palatability (Gońi et al. 2007).

The impact of ageing process on the quality of beef has already been investigated in many experiments, taking into account different ageing time. Since with a longer ageing time, a higher cost must be borne by the beef producer, it would be beneficial to use 9-day instead of 14-day ageing in the production of high quality culinary beef, also from dairy breeds. There are reports in which the effect of ageing up to the 14th day was investigated, but the sampling days ranged from the 1st, 2nd or 3rd to 7th or 14th day of ageing (Beriain et al. 2009; Domaradzki et al. 2017; Li et al. 2011), which affected the results of statistical analyses, indicating the effect of ageing on meat quality. Therefore, it was difficult to draw conclusions about the effect of ageing time between the 9th and 14th day of ageing based on previous studies.

Herbs and herbal preparations (which are a source of bioactive compounds) in cattle feeding might be used for improving digestion process, appetite stimulation, to alleviate stress (www.farmwet.pl) and to improve the quality of meat (de Zawadzki et al. 2017). The examples of commercially available preparations are Optirum, which contains extracts of 12 herbs (the composition provided in “Experimental groups and feeding of animals” section) and live yeast cultures, recommended to improve the functioning of the gastrointestinal tract of cattle and to stimulate appetite, and Stresomix, which contains extracts of 8 herbs and is recommended to reduce stress, increase the functioning of the immune system and facilitate adaptation (www.farmwet.pl). Although herbal preparations for animal nutrition are commercially available, there is still a shortage of papers indicating their influence on beef quality. Modzelewska-Kapituła et al. (2018) noted the influence of Optirum and Stresomix on the quality of bovine *longissimus lumborum* and *semimembranosus* muscles and the significant positive effect on the tenderness of *longissimus lumborum* muscle after 14-days ageing. Taking into consideration that *longissimus lumborum* muscle is commonly used for steaks and consumed after short-time thermal treatment, the tenderness of the cut is a vitally important quality attribute from the consumer perspective. Due to the fact that the impact of herbal preparations on meat quality was widely discussed in previous paper, the aim of this study was to investigate the changes in the quality of *longissimus lumborum*, obtained from dairy cattle fed with different dietary treatments, that occur between 9th and 14th day of ageing, and to test the hypothesis that adding herbal preparations to Holstein-Friesian bulls’ feed decreases ageing time without a negative effect on beef quality.

## Materials and methods

### Experimental groups and feeding of animals

The experiment was conducted using twenty-four Polish Holstein-Friesian bulls. Starting from 6th month of age, the bulls were fattened semi-intensively, receiving ad libitum a total mixed ration (TMR) composed of maize silage with the addition of 2 kg concentrate. When the animals reached a body weight of about 430 kg (age approx. 15 months), they were stratified into three groups (n = 8 individuals in each group) according to weight (the analogue method). The bulls were housed in a monitored-feeding building on deep bedding in three separate pens. C group received a control (basal) diet, O group received a basal diet with Optirum preparation and OS group received a basal diet with Optirum and Stresomix preparations. Chemical composition and nutritional value of diets were presented in Table 1. Animals from each group were fed ad libitum a total mixed ration (TMR) composed of maize silage and 3.5 kg concentrate. The concentrate contained rapeseed meal 15%, triticale meal 82.5% and premix 2.5% (code of product 7619; Cargill Poland Ltd., Warsaw, Poland; Ca, 235 g; Na, 79 g; P, 48 g; Mg, 28 g; Fe, 500 g; Mn, 2000 mg; Cu, 375 mg; Zn, 3750 mg; J, 50 mg; Co, 12.5 mg; Se, 12.50 mg; vitamin A, 250,000 IU; vitamin D3, 50,000 IU; vitamin E, 1000 mg; dl-alpha-tocopherol, 909.10 mg per kg). There was a 2-week adaptation period before the final fattening of the bulls from O and OS groups began. The fattening with the fodder containing an addition of the preparations lasted 5 months. TMR was administered from a self-propelled feed cart (Seko, Curtarolo, Italy) and delivered to feeding stations twice daily (at 08:00 and 16:00 h). The control group was fed exclusively TMR, the O group received also Optirum (20 g/animal/day), whereas OS group was fed TMR with Optirum (20 g/animal/day) and in the last month of feeding also Stresomix (40 g/animal/day). Optirum and Stresomix were blended with premix, which was then placed in the concentrate with the remaining components. The following ingredients were included in the Optirum preparation: *Trigonella foenum graecum*, *Woodfordia fruticosa*, *Andrographis paniculata*, *Phyllanthus emblica*, *Terminalia belerica*, *Terminalia chebula*, *Coriandrum sativum*, *Allium sativum*, *Balanites roxburghii*, *Curcuma longa*, *Zingiber officinale*, *Semecarpus anacardium* herbal extracts and live yeast cultures, whereas Stresomix was composed of *Ocimum sanctum*, *Withania somnifera*, *Phyllanthus emblica*, *Asparagus racemosus*, *Glycyrrhiza glabra*, *Tribulus terrestris*, *Mangifera indica*, *Shilajit* herbal extracts (www.farmwet.pl). Optirum is recommended for improving fattening efficiency and meat quality, while Stresomix should be administered during the last month before slaughter to reduce the stress of animals during transport and slaughter (www.farmwet.pl). During the fattening, the average daily gain of the bulls weight was 1.3 kg. The fodder intake of each bull was controlled individually using the Roughage Intake Control System (Insentec BV, Marknesse, The Netherlands). When the animals reached 600 kg of body weight, the fattening was finished. The bulls were slaughtered on two different occasions (n = 12 per session) in compliance with Council Regulation (EC) No 1099/2009 of 24 September 2009 on the protection of animals at the time of killing. After the bulls were delivered to an abattoir, they were fasted for 15 to 20 h in individual boxes with free access to water. Before and after slaughter the animals were weighed with an accuracy of 0.5 kg.

**Table 1:** Composition and nutritional value of control, Optirum (O treatment) and Optirum and Stresomix supplemented (OS treatment) diets

Specification	Control	O treatment	OS treatment
TMR	Ad libitum	Ad libitum	Ad libitum
Optirum (g)	–	20.0	20.0
Stresomix (g)	–	–	40 (last month of feeding)
Chemical composition and nutrition value (g/kg DM) diets			
Dry matter (g/kg)	472.6	473.3	474.8
Organic substance (g)	938.3	938.3	938.4
Total protein (g)	119.7	119.8	119.9
Crude fat (g)	24.6	24.6	24.6
Crude fibre (g)	163.7	163.9	164.2
UFV^a^	0.92	0.90	0.90
PDIN^c^	77.2	77.3	77.6
PDIN^b^	90.9	91.0	91.2

TMR (Total Mixed Ration) (TMR) composed of maize silage and 3.5 kg concentrate (rapeseed meal 15%, triticale meal 82.5% and premix 2.5%); NNH_3_/N total 64.3 g/kg; pH 3.92

*DM* dry matter

^a^Meat fodder units

^b^Protein digested in the small intestine when rumen fermentable N is limiting

^c^Protein digested in the small intestine when rumen fermentable energy is limiting

### Muscle sampling

Muscles *longissimus lumborum* (LL, n = 24) were removed from left half-carcasses of each animal 24 h post mortem and then transported (about 3 h) to a laboratory in a portable fridge cooler at refrigerated temperature. The muscles were placed into a refrigerator (4 ± 1 °C) and kept overnight. After that, each muscle was cut into two parts (one for 9-day and another for 14-day ageing, approx. 800 g each), which were individually vacuum-packed in PA/PE (Inter Arma sp. z o.o., Rudawa, Poland; thickness 70 µm, total transmission rates not exceeding 10 mg/dm^2^ for model liquids, 3% acetic acid, 50% ethyl alcohol for 10 days at 40 °C and isooctane for 2 days, 20 °C). The samples were stored until the 9th and 14th day post mortem at 4 ± 1 °C (Memmert GmbH, Schwabach, Germany) and the meat was then removed from the vacuum, dabbed dry with a paper towel and weighed to enable determination of purge loss. A 300 g sample was allocated for physico-chemical analyses, whereas two 2.54 cm-thick steaks (average weight 214 g ± 30 g) were cut to determine sensory quality, cooking loss, and Warner–Bratzler Shear Force (WBSF).

### Raw meat quality assessment

After 9 and 14 days post mortem, the samples were analysed for pH value, moisture and expressible water contents and colour. The external fat and thick connective tissue were removed from the muscles and the meat was ground twice using a 3 mm mesh and then thoroughly manually mixed. pH was measured directly in the ground meat with a combined electrode FC 200 and pH-meter HI 8314 (Hanna Instruments Polska, Olsztyn, Poland). The devise was first calibrated using pH 7 and pH 4 buffers. Moisture was determined according to PN-ISO^1442^ (2000) (dying at 103 ± 2 °C to a constant weight,) and the expressible water content according to Hamm (1986) with modification (Modzelewska-Kapituła et al. 2018). The content of expressible water was expressed in % in respect to the sample weight and in % of moisture.

To determine raw meat colour in the CIE L*a*b* system, a MiniScan XE Plus device (HunterLab, Reston, USA) with standard illuminant D65, a 10° standard observer angle and a 2.54-cm-diameter aperture was used. The colorimeter was calibrated before colour determination using white and black tiles supplied by the manufacturer. Colour was determined on fresh cut steaks, which were left on trays to refrigerate (4 ± 1 °C) for 1 h to allow blooming. The indexes of hue angle (H) of the samples H = arctangent (b*/a*)·360°/(2·Π) and chroma (C) C = (a*^2^ + b*^2^)^0.5^ were calculated. All analyses were performed in triplicate.

### Thermal treatment and cooked meat quality assessment

Thermal treatment was proceeded in a preheated convectional-steam oven (Retigo Vision 623i, Retigo, Rožnov pod Radhoštěm, Czech Republic). The steaks were heated in 100% steam environment at 100 °C to obtain 75 °C. A thermometer connected to the oven was used to monitor the internal temperature of the steaks. After the cooking following attributes were determined: cooking loss, Warner–Bratzler Shear Force and the sensory quality. To determine cooking loss, the steaks were weighed before and after cooking.

WBSF values (N) were measured on the samples (10 mm × 10 mm, about 40 mm long, n = 5 from each steak) cut from cooked and then chilled (3 ± 1 °C) steaks, parallel to the longitudinal orientation of the muscle fibres, using Instron 5942 (Instron, Norwood, USA) equipped with a shear blade with a triangular aperture of 60° (load 500 N, head speed 200 mm/min). The samples at room temperature (approximately 20 °C) were cut perpendicular to the longitudinal orientation of the muscle on meat. Bluehill 3 software (Instron, Norwood, USA) was used.

Immediately after the termination of the thermal treatment, samples were cut into the approximately 2 mm thick slices, coded with three-digit numbers and served to the panellists (n = 6, trained for 36 h, non-smokers, females) randomly on white plates. Panellists scored each sample for meat and atypical aroma (1, imperceptible; 10, extremely intense), juiciness (1, extremely dry; 10, extremely juicy), tenderness (1, extremely tough; 10, extremely tender), meat and atypical taste (1, imperceptible; 10, extremely intense). In total, 4 sensory analysis sessions were performed during which a maximum of 6 meat samples were evaluated. The sessions were carried out at room temperature (approx. 20 °C) under fluorescent lighting. Water and bread were used to cleanse the palate. At the same time, 3 samples were presented followed by an approx. 20 min interval before the assessment of the next samples.

### Data analysis

Statistical analysis of the gathered data was performed using Statistica 12 (StatSoft Inc., Tulsa, OK., USA) software. The results were presented as mean values and standard deviations. To examine the differences between mean values, excluding sensory analysis results, an analysis of variance was conducted along with Duncan’s test. To compare sensory analysis results, non-parametric U Mann–Whitney and Kruskal–Wallis tests were applied to compare two and more groups of means, respectively. The significance level was set at *P* < 0.05. A two-way Anova was applied to determine the effect of ageing (two levels: 9 and 14 days) and dietary treatment (three levels: control, O, OS) on the quality of beef.

## Results

### Animal performance

At the end of the fattening, the Polish Holstein-Friesian bulls obtained 598.1 (± 18.8), 601.2 (± 20.5) and 591.1 (± 0.8) kg of body weight, for control, O and OS treatments, respectively (*P* > 0.05). There were no differences in age at slaughter between the treatments (20.5 ± 0.7, 20.8 ± 0.8 and 20.2 ± 0.8 months for control, O and OS treatments, respectively) nor in hot carcass weight (318.3 ± 10.6, 323.6 ± 9.6 and 315.5 ± 10.1 kg for control, O and OS treatments, respectively). The carcasses also showed similar (*P* > 0.05) dressing percentage (53.2 ± 0.4, 53.8 ± 0.7 and 53.4 ± 0.6% for control, O and OS treatment, respectively). The dressing percentages of the carcasses were in the range of variation considered adequate (from 52.1 to 56.4%) for Holstein-Friesian bulls at similar ages (Nogalski et al. 2013, 2014; Węglarz 2011). The results of this study are in agreement with the findings of de Zawadzki et al. (2017), who also reported a lack of differences in animal performance between bulls fed control and plant extract (mate, up to 1.5%) supplemented diets.

### pH

The mean values of pH (5.52 to 5.77, Table 2) noted in the present study indicated normal quality of beef. Between 9th and 14th day of ageing, the pH values remained unchanged for O treatment, whereas a decrease in pH for control treatment and an increase for OS treatment were noted. However, two-way Anova revealed that the effect of ageing on beef pH was insignificant, in contrast to the effect of dietary treatment: the lowest pH was found for the control, whereas the highest was found for OS treatment (Table 3). The results resembled, to some extent, those of Franco et al. (2012), who reported that the pH value of beef steaks was significantly affected by ageing time and diet. Kim et al. (2010) also reported no changes in pH with storage time. On the other hand, Leheska et al. (2008) did not find any differences in pH between beef from control and grass-fed treatments.

**Table 2:** Comparison of quality attributes of raw and steam-cooked beef originated from bulls (n = 8 per treatment) fed with different diets, after 9 and 14-day ageing (mean values ± standard deviation)

Parameter	Control		O treatment		OS treatment	
	9 days	14 days	9 days	14 days	9 days	14 days
Raw beef						
pH	5.64 ± 0.05^b^	5.52 ± 0.03^c^	5.63 ± 0.04^b^	5.62 ± 0.14^b^	5.65 ± 0.04^b^	5.77 ± 0.08^a^
Moisture (%)	73.95 ± 1.27^a^	73.89 ± 2.00^a^	73.92 ± 1.73^a^	73.43 ± 1.15^a^	74.44 ± 1.15^a^	73.21 ± 1.68^a^
Expressible water (%)	32.68 ± 2.50^a^	29.47 ± 2.90^b^	30.48 ± 3.46^b^	26.03 ± 3.63^d^	27.78 ± 1.84^c^	21.50 ± 2.76^e^
Expressible water (% of moisture)	44.18 ± 3.14^a^	39.95 ± 4.32^b^	41.22 ± 4.50^b^	35.42 ± 4.74^c^	37.33 ± 2.61^c^	29.39 ± 3.87^d^
Purge loss (%)	2.78 ± 2.75^ab^	3.54 ± 1.56^a^	1.89 ± 1.30^ab^	3.11 ± 2.01^a^	0.95 ± 0.30^b^	1.78 ± 0.74^ab^
Lightness, L*	36.98 ± 2.01^a^	37.77 ± 2.09^a^	35.56 ± 1.33^b^	35.80 ± 1.35^b^	37.41 ± 2.29^a^	37.01 ± 1.92^a^
Redness, a*	19.87 ± 1.70^ab^	20.45 ± 1.98^a^	19.10 ± 2.13^bc^	20.70 ± 1.22^a^	18.70 ± 1.37^c^	20.03 ± 1.53^ab^
Yellowness, b*	15.24 ± 1.44^abc^	15.88 ± 1.57^a^	14.56 ± 1.55^c^	15.39 ± 0.85^ab^	14.80 ± 1.38^bc^	15.91 ± 1.05^a^
Hue angle, H	52.55 ± 1.78^ab^	52.18 ± 1.83^ab^	52.65 ± 3.28^b^	53.39 ± 0.80^a^	51.70 ± 2.16^b^	51.54 ± 1.57^ab^
Chroma, C	25.05 ± 2.08^ab^	25.91 ± 2.40^a^	24.05 ± 2.29^bc^	25.79 ± 1.44^a^	23.86 ± 1.74^c^	25.59 ± 1.72^a^
Steam-cooked beef						
Cooking loss (%)	29.24 ± 4.21^ab^	30.91 ± 4.60^a^	27.26 ± 3.60^ab^	25.89 ± 4.78^bc^	28.76 ± 1.57^ab^	23.28 ± 2.27^c^
WBSF (N)	75.52 ± 4.62^a^	58.14 ± 15.80^b^	69.82 ± 9.16^a^	71.04 ± 21.29^a^	57.53 ± 7.11^b^	46.16 ± 16.68^c^

Control—basal diet; O—basal diet plus Optirum; OS—basal diet plus Optirum and Stresomix

Optirum: *Trigonella foenum graecum*, *Woodfordia fruticosa*, *Andrographis paniculata*, *Phyllanthus emblica*, *Terminalia belerica*, *Terminalia chebula*, *Coriandrum sativum*, *Allium sativum*, *Balanites roxburghii*, *Curcuma longa*, *Zingiber officinale*, *Semecarpus anacardium;* Stresomix: *Ocimum sanctum*, *Withania somnifera*, *Phyllanthus emblica*, *Asparagus racemosus*, *Glycyrrhiza glabra*, *Tribulus terrestris*, *Mangifera indica*, *Shilajit*

^a,b,c^Mean values in a row with different letters differ at *P* < 0.05

**Table 3:** Influence of ageing time (A) and dietary treatment (D) on quality attributes of *longissimus lumborum* (mean values ± standard deviation)

Parameter	Ageing (A)		Dietary treatment (DT)			Significance (P value)		
	9 days	14 days	Control	O	OS	A	DT	A × DT
Raw beef								
pH	5.64 ± 0.04^x^	5.64 ± 0.14^x^	5.58 ± 0.07^c^	5.63 ± 0.10^b^	5.71 ± 0.09^a^	NS	0.000	NS
Moisture (%)	74.10 ± 1.41^x^	73.51 ± 1.65^y^	73.92 ± 1.66^a^	73.67 ± 1.47^a^	73.82 ± 1.55^a^	0.021	NS	NS
Expressible water (%)	30.31 ± 3.33^x^	25.67 ± 4.50^y^	31.08 ± 3.13^a^	28.25 ± 4.17^b^	24.64 ± 3.93^c^	0.0000	0.0000	0.036
Expressible water (% of moisture)	40.91 ± 4.46^x^	34.92 ± 6.10^y^	42.06 ± 4.31^a^	38.32 ± 5.43^b^	33.36 ± 5.17^c^	0.0000	0.0000	NS
Purge loss (%)	1.88 ± 1.87^x^	2.78 ± 1.65^x^	3.14 ± 2.23^a^	2.54 ± 1.77^ab^	1.37 ± 0.70^b^	NS	0.016	NS
Lightness, L*	36.65 ± 2.05^x^	36.86 ± 1.97^x^	37.37 ± 2.06^a^	35.68 ± 1.33^b^	37.21 ± 2.10^a^	NS	0.0000	NS
Redness, a*	19.22 ± 1.80^y^	20.39 ± 1.61^x^	20.16 ± 1.85^a^	19.90 ± 1.90^a^	19.37 ± 1.59^a^	0.0000	NS	NS
Yellowness, b*	14.86 ± 1.47^y^	15.73 ± 1.20^x^	15.56 ± 1.53^a^	14.97 ± 1.30^a^	15.35 ± 1.34^a^	0.0000	NS	NS
Hue angle, H	52.30 ± 2.49^x^	52.37 ± 1.64^x^	52.36 ± 1.79^ab^	53.02 ± 2.39^a^	51.62 ± 1.87^b^	NS	0.004	NS
Chroma, C	24.32 ± 2.09^y^	25.76 ± 1.87^x^	25.48 ± 2.26^a^	24.92 ± 2.09^a^	24.72 ± 1.92^a^	0.0000	NS	NS
Steam-cooked beef								
Cooking loss (%)	28.42 ± 3.29^x^	26.69 ± 5.04^x^	30.08 ± 4.34^a^	26.58 ± 4.15^b^	26.02 ± 3.40^b^	NS	0.007	0.031
WBSF (N)	68.98 ± 10.02^x^	59.36 ± 20.26^y^	66.93 ± 14.45^a^	70.40 ± 15.97^a^	51.95 ± 13.86^b^	0.0000	0.0000	0.0000

Control—basal diet; O—Optirum treatment; OS—Optirum + Stresomix treatment

Optirum: *Trigonella foenum graecum*, *Woodfordia fruticosa*, *Andrographis paniculata*, *Phyllanthus emblica*, *Terminalia belerica*, *Terminalia chebula*, *Coriandrum sativum*, *Allium sativum*, *Balanites roxburghii*, *Curcuma longa*, *Zingiber officinale*, *Semecarpus anacardium;* Stresomix: *Ocimum sanctum*, *Withania somnifera*, *Phyllanthus emblica*, *Asparagus racemosus*, *Glycyrrhiza glabra*, *Tribulus terrestris*, *Mangifera indica*, *Shilajit*

*NS* non-significant

^x,y^Mean values in a row with different letters within ageing differ at *P* < 0.05

^a,b,c^Mean values in a row with different letters dietary treatment differ at *P* < 0.05

### Moisture and water holding capacity

Moisture and water holding capacity (WHC), including expressible water content (% and % of moisture), purge loss (%) and cooking loss (%) of beef originated from bulls fed with different dietary treatments, after 9-days and 14-days wet ageing, are shown in Table 2. Moisture content in raw beef was similar in all treatments (approx. 74%). For all dietary treatments, lower expressible water content (% and % of moisture) was noted for 14-days aged beef compared with those after 9-days ageing. There were no differences in the purge loss (%) values noted on different sampling days within each treatment. Only in the case of OS treatment was there a significant decrease in cooking loss between the 9th and 14th day of ageing.

The influence of ageing and dietary treatment on WHC is presented in Table 3. Ageing affected moisture (*P* < 0.05) and expressible water (*P* < 0.001) contents, which decreased over time. On the other hand, dietary treatment affected expressible water contents (*P* < 0.001), purge loss (*P* < 0.05) and cooking loss (*P* < 0.01). Moreover, there was an interaction between ageing and dietary treatment for expressible water content and cooking loss (*P* < 0.05) (Table 3). The results obtained related to water holding capacity of beef (moisture content, expressible water content, purge and cooking loss) were similar to those noted in earlier studies on beef quality (Muchenje et al. 2009). Although the differences in purge loss between 9th and 14th day of ageing were insignificant, they tended to increase in time and could have resulted in the reduction of moisture content in the muscles. This, in turn, could have reduced cooking loss and WBSF values, due to the fact that a higher level of moisture in beef can lead to higher cooking loss and lower tenderness (Chambaz et al. 2003). An increased cooking loss has a negative effect on beef tenderness (Silva et al. 1999). Although in the present study there were no significant differences in cooking loss between 9-days and 14-days aged beef, lower values of cooking loss corresponded to lower moisture content and WBSF values.

Lower values of expressible moisture found in 14-days aged beef indicate that the water holding capacity of meat increased. Kristensen and Purslow (2001) explained this phenomenon by degradation of cytoskeletal proteins and removal of the force that makes the moisture flow into extracellular space. Farouk et al. (2012) introduced the term “sponge effect” for the phenomenon, which occurs during meat ageing and consists in the fact that the canals in meat structure, through which moisture is expelled, are distracted and free water is physically entrapped in meat, which reduces the amount of water that drips out. Although there were no differences in moisture between dietary treatments, the best WHC (the lowest expressible water content) was found in beef from OS treatment, which corresponded with the highest meat pH values. The samples also showed lower purge and cooking loss than the control treatment. This could be explained by the fact that along with an increase in meat pH value, the WHC increases due to a higher number of overall negative charge of proteins, which causes filament repulsion and creates more space for water molecules (Huff-Lonergan and Lonergan 2005). The findings of the present study also highlight the effect of the dietary treatment in obtaining good technological properties of beef, taking into consideration that purge and cooking losses affect production yields and, thus, production profitability.

### Colour

Lightness (L*) of the beef ranged from 35.6 to 37.8, redness (a*) from 18.7 to 20.7 and yellowness (b*) from 14.6 to 15.9 (Table 2). Ageing time did not produce changes in L* within each dietary treatments. It was noted that beef from O treatment showed significantly lower values than beef from control and OS treatments. Between 9^th^ and 14th day of ageing, a* and b* values and chroma increased significantly in beef from O and OS treatments. Hue angle increased only in beef from the O treatment in time, whereas in the remaining treatments no changes were noted. Ageing time affected redness, yellowness and chroma (*P* < 0.000), which significantly increased between 9th and 14th day of ageing, whereas lightness and hue angle were affected (*P* < 0.000 and *P* < 0.01, respectively) by dietary treatment (beef from O treatment was characterized by lower L* values that control and OS treatments, and higher H than OS treatment) (Table 3). A significant effect of dietary treatment on lightness and hue angle was also reported by Ripoll et al. (2013) in veal and by de Zawadzki et al. (2017) and Franco et al. (2012) in beef.

Similar values of colour parameters L* and a* were obtained in previous studies and shown in the review by Muchenje et al. (2009). However, in the present study, higher values of yellowness (regardless of dietary treatment and ageing time) were reported compared with earlier studies (6.1–11.3) (Muchenje et al. 2009) and were lower than those shown by Canto et al. (2015) (27.1–28.6). Although there are many reports in which the effect of ageing on beef colour was discussed, different ageing times have been studied, such as 24 h, 2-days, 3-days and 7-days (Li et al. 2011), 48 h, 7-days and 14-days (Domaradzki et al. 2017) or 3-days, 7-days and 14-days (Beriain et al. 2009). In all of these studies, ageing significantly affected a* and b* values, which increased in time, which was also noted in this study. Comparing the results obtained for similar ageing times, Domaradzki et al. (2017) noted a decrease in L*, and no changes in a* and b* values in beef from Polish Holstein-Friesian bulls between the 7th and 14th day of ageing. These results, except for L*, resemble those obtained in the present study for control treatment. Beriain et al. (2009) also reported a lack of changes in L*, a*, b*, C and H between the 7th and 14th day of ageing. The differences in the results obtained in the present study and those of Beriain et al. (2009) might result from different cattle breed and dietary treatments, due to the fact that increases in a*, b* and C were noted in the present study only for treatments in which herbal extracts were used as dietary additives, but not for the control. The increase in a* and b* values in beef during ageing might be explained by the decreased activity of mitochondria, which results in higher oxygen level on the beef surface and its higher diffusion. Larger quantities of oxygen available for myoglobin causes its oxygenation, which manifests in more red and yellow hues and increased saturation (Beriain et al. 2009). Since this phenomenon was noted in beef from dietary treatments containing herbal extracts, it might indicate that bioactive substances present in the diets inhibited myoglobin oxidation and metmyoglobin formation in beef. Moreover, the changes presumably made the beef aged for 14 days more attractive for consumers, due to more red and more saturated colour, as far as there is a positive correlation between a* and the acceptability of beef colour by consumers (Beriain et al. 2009).

### Warner–Bratzler Shear Force

The Warner–Bratzler Shear Force values ranged from 46.2 N (14-days aged OS treatment) to 75.5 N (9-days aged control) (Table 2). Between 9th and 14th day of ageing, WBSF values significantly decreased in beef from the control and OS treatment. However, in beef from O treatment, no differences in WBSF values over time were noted, which might indicate that ageing in beef from this treatment processed with different rates (presumably slower) than in the control and OS treatments. Moreover, 14-days aged beef from OS treatment had the lowest (*P* < 0.05) WBSF values (Table 2). For WBSF, both factors (ageing time and dietary treatment) affected the results (*P* < 0.000) and significant interaction between the factors was noted (*P* < 0.000) (Table 3). Generally, WBSF values decreased between the 9th and 14th day of ageing, which was expected. The lowest WBSF values were found in beef from OS treatment, which indicated the best tenderness of the meat (Table 3). The effect of the dietary treatment on WBSF values could be explained by the possible increase in antioxidants in meat (e.g. carnosine), which may inhibit protein oxidation, which results in more tender meat (de Zawadzki et al. 2017). The effect of dietary treatment (mate extract addition) on WBSF of beef was found also by de Zawadzki et al. (2017), who noted that non-supplemented bulls meat (control) had significantly higher values of WBSF. Similar to the present study, Ripoll et al. (2013) also reported a significant effect of the production system, ageing and interaction between the factors on WBSF of veal. Taking into consideration the WBSF values, Destefanis et al. (2008) classified beef as very tender with WBSF below 32.96 N, tender with WBSF from 32.96 to 42.77 N, acceptably tender with WBSF from 42.87 to 52.68 N, hard with WBSF from 52.78 to 62.59 N and very hard with WBSF above 62.59 N. According to this classification, 9-days aged beef from the control treatment and 9-days and 14-days aged beef from O treatments were very hard, 14-days aged beef from the control treatment and 9-days aged beef from OS treatment were hard, whereas 14-days aged beef from OS treatment was acceptably tender. Generally, beef from dairy cattle had higher WBSF values than from beef breeds. Bureš and Bartoň (2018) found that LL from Holstein bulls had significantly higher WBSF values than from Aberdeen Angus (58.6 vs. 36.0 N/cm^2^). The significant decrease in WBSF during ageing has already been well-documented and was also reported by Węglarz (2010), who found that between the 7th and 14th day of ageing, WBSF decreased from 6.64 (65.12 N) to 3.77 kG/cm^2^ (36.97 N) in LL muscle obtained from Polish Holstein-Friesian bulls fattened semi-intensively to 550 kg of final body weight. On the other hand, the results of the present study, which show an insignificant decrease in WBSF for O treatment, resemble those reported by Domaradzki et al. (2017), who reported that WBSF values for LL originating from 18-month-old Polish Holstein-Friesian bulls were 69.3 N and 65.3 N after 7-days and 14-days ageing, respectively.

### Sensory quality

The sensory quality of the samples was evaluated on a 10-point scale and the results are shown in Table 4. The attributes, which gained low scores, were the intensity of untypical aroma and untypical taste (below 1.4 points), which indicates that aroma and taste of the samples originated from different dietary treatments and aged for different time, were typical for good quality beef. The tenderness of the samples was scored in the range from 3.3 (9-day aged O treatment) to 5.5 points (14-day aged OS treatment). Scores for intensity of meat aroma and taste gained relatively high scores, up to 7.4 points. The only change in the scores in time of ageing was noted for OS treatment in intensity of meat aroma, whereas in the remaining treatments there were no differences between the results noted after 9 and 14 days of ageing. However, when two-way Anova was applied, it was shown that ageing time affected the intensity of meat aroma (*P* ≤ 0.05) and tenderness (*P* ≤ 0.001), which were scored higher after 14-day ageing and intensity of untypical taste (*P* ≤ 0.05), which decreased during ageing (Table 5). This indicates that the eating quality of beef improved along with the ageing time (tenderness, intensity of meat aroma, typical taste). Moreover, the increase in sensory evaluated tenderness between 9th and 14th day of ageing corresponded to a decrease in WBSF values. Dietary treatment affected the intensity of meat aroma (control and O samples were scored higher than O treatment, *P* ≤ 0.001), tenderness (OS samples gained higher scores than O and control, *P* ≤ 0.001) and intensity of meat taste (the control was scored higher than OS treatment, *P* ≤ 0.01).

**Table 4:** Comparison of sensory quality of steam-cooked beef originated from bulls (n = 8 from each treatment) fed with different diets, after 9 and 14 day ageing (mean values ± standard deviation)

Parameter	Control		O treatment		OS treatment	
	9 days	14 days	9 days	14 days	9 days	14 days
Intensity of meat aroma	6.79 ± 1.71^abc^	7.32 ± 1.78^a^	6.78 ± 1.59^abc^	7.11 ± 1.78^ab^	5.87 ± 1.49^c^	6.29 ± 1.81^b^
Intensity of untypical aroma	1.19 ± 0.50^a^	1.21 ± 0.60^a^	1.25 ± 0.54^a^	1.05 ± 0.23^a^	1.18 ± 0.39^a^	1.03 ± 0.17^a^
Juiciness	5.25 ± 1.79^a^	5.83 ± 2.04^a^	5.18 ± 2.01^a^	5.13 ± 1.83^a^	5.41 ± 1.67^a^	5.34 ± 1.37^a^
Tenderness	3.38 ± 1.75^b^	4.45 ± 2.07^ab^	3.28 ± 1.58^b^	4.39 ± 2.06^ab^	4.92 ± 1.86^a^	5.49 ± 1.72^a^
Intensity of meat taste	7.17 ± 1.43^a^	7.45 ± 1.37^a^	6.80 ± 1.83^a^	6.82 ± 1.84^a^	6.51 ± 1.78^a^	6.60 ± 1.70^a^
Intensity of untypical taste	1.38 ± 0.84^a^	1.19 ± 0.52^a^	1.35 ± 0.77^a^	1.11 ± 0.39^a^	1.18 ± 0.56^a^	1.09 ± 0.28^a^

Control—basal diet; O—basal diet plus Optirum; OS—basal diet plus Optirum and Stresomix

Optirum: *Trigonella foenum graecum*, *Woodfordia fruticosa*, *Andrographis paniculata*, *Phyllanthus emblica*, *Terminalia belerica*, *Terminalia chebula*, *Coriandrum sativum*, *Allium sativum*, *Balanites roxburghii*, *Curcuma longa*, *Zingiber officinale*, *Semecarpus anacardium;* Stresomix: *Ocimum sanctum*, *Withania somnifera*, *Phyllanthus emblica*, *Asparagus racemosus*, *Glycyrrhiza glabra*, *Tribulus terrestris*, *Mangifera indica*, *Shilajit*

^a,b,c^Mean values in a row with different letters differ at *P* < 0.05

**Table 5:** Influence of ageing time (A) and dietary treatment (D) on sensory quality of *longissimus lumborum*

Parameter	Ageing (A)		Dietary treatment (DT)			Significance (P value)		
	9 days	14 days	control	O	OS	A	DT	DT × A
Intensity of meat aroma	6.50 ± 1.65^y^	6.97 ± 1.83^x^	7.07 ± 1.76^a^	6.94 ± 1.69^a^	6.07 ± 1.65^b^	0.050	0.001	NS
Intensity of untypical aroma	1.21 ± 0.48^x^	1.11 ± 0.42^x^	1.20 ± 0.55^a^	1.15 ± 0.43^a^	1.11 ± 0.31^a^	NS	NS	NS
Juiciness	5.28 ± 1.82^x^	5.48 ± 1.82^x^	5.55 ± 1.94^a^	5.15 ± 1.91^a^	5.38 ± 1.52^a^	NS	NS	NS
Tenderness	3.83 ± 1.87^y^	4.72 ± 2.02^x^	3.95 ± 1.9^b^	3.82 ± 1.91^b^	5.19 ± 1.80^a^	0.000	0.000	NS
Intensity of meat taste	6.85 ± 1.68^x^	7.02 ± 1.65^x^	7.32 ± 1.40^a^	6.81 ± 1.82^ab^	6.55 ± 1.73^b^	NS	0.009	NS
Intensity of untypical taste	1.31 ± 0.74^x^	1.13 ± 0.43^y^	1.28 ± 0.69^a^	1.23 ± 0.62^a^	1.14 ± 0.45^a^	0.024	NS	NS

Control—basal diet; O—Optirum treatment; OS—Optirum + Stresomix treatment

Optirum: *Trigonella foenum graecum*, *Woodfordia fruticosa*, *Andrographis paniculata*, *Phyllanthus emblica*, *Terminalia belerica*, *Terminalia chebula*, *Coriandrum sativum*, *Allium sativum*, *Balanites roxburghii*, *Curcuma longa*, *Zingiber officinale*, *Semecarpus anacardium;* Stresomix: *Ocimum sanctum*, *Withania somnifera*, *Phyllanthus emblica*, *Asparagus racemosus*, *Glycyrrhiza glabra*, *Tribulus terrestris*, *Mangifera indica*, *Shilajit*

*NS* non-significant

^x,y^Mean values in a row with different letters within ageing differ at *P* < 0.05

^a,b,c^Mean values in a row with different letters dietary treatment differ at *P* < 0.05

Similar results were reported by Bruce et al. (2005), who found that ageing did not affect beef juiciness and concluded that juiciness is determined within the first 3 days post-mortem and changes only slightly during ageing up to 14 day post-mortem. They showed also the improvement in tenderness for most of studied beef in the time of 14-days ageing (Bruce et al. 2005). Changes in the intensity of meat aroma in ageing time in beef results from the formation of numerous volatile compounds, which affect the aroma of raw and cooked meat (Colle et al. 2015). However, their influence on meat quality can be different. Resconi et al. (2018) showed that the intensity of the typical aroma of raw beef decreased with ageing. Gorraiz et al. (2002), who studied the changes in flavour volatiles in beef during the first 7 days post-mortem ageing, noted that the characteristic flavour and aftertaste of cooked meat increased. Moreover, the final flavour of cooked meat results from a wide variety of volatile compounds, which are formed during heat treatment, mainly in Maillard reactions and lipid degradation (Gorraiz et al. 2002). Thus, another factor, which might affect beef aroma is intramuscular fat content. However, in the present study, this factor had no influence on the results since the fat content in 14-days aged LL muscles did not differ between dietary treatments (Modzelewska-Kapituła et al. 2018). In the present study, the intensity of meat aroma and taste, but not intensity of untypical aroma and taste, were affected by the dietary treatment. Similar results were reported by de Zawadzki et al. (2017), who found that mate extract addition (1.5%) to the bulls’ feed decreased the intensity of characteristic beef aroma but had no influence on either foreign aroma or foreign flavour intensity of the meat.

## Conclusion

The quality of beef obtained from Holstein-Friesian bulls depended on beef ageing time and dietary treatment. The results of this study demonstrate marked differences in the ageing process in meat obtained from the carcasses of the bulls fed with different dietary treatments. Since 14-days aged beef showed more red and saturated colour, lower WBSF and sensorial tenderness and more intense meat aroma than 9-days aged meat, a longer ageing time should be applied to obtain good quality. However, using two herbal preparations (Optirum and Stresomix) in the dietary treatment enabled to obtain beef with similar tenderness after 9-days ageing compared with 14-days aged beef from the control treatment. This indicates that using a well-designed dietary treatment plan decreases the ageing time of beef.
